# Molecular and phenotypic reidentification of *Sporothrix schenckii* clinical isolates preserved under mineral oil for 34 to 64 years in a culture collection in Brazil

**DOI:** 10.1016/j.crmicr.2022.100128

**Published:** 2022-03-24

**Authors:** Thais Barreira, Danielly Corrêa-Moreira, Cintia Borba, Aurea Moraes, Manoel Oliveira

**Affiliations:** a Laboratory and Facility Multi-User, Evandro Chagas National Institute of Infectious Diseases, FIOCRUZ, Rio de Janeiro, Brazil; b Clinical Research in Infectious Diseases, Evandro Chagas National Institute of Infectious Diseases, FIOCRUZ, Rio de Janeiro, Brazil; c Laboratory of Taxonomy, Biochemistry and Bioprospecting of Fungi, Oswaldo Cruz Institute, FIOCRUZ, Rio de Janeiro, Brazil; d International Platform for Science , Technology and Innovation in Health

**Keywords:** Culture collection, Viability, Morphophysiological stability, Molecular identification and β-tubulin, Polyphasic identification, *Sporothrix schenckii*, Sporotrichosis

## Abstract

Sporotrichosis is a subcutaneous mycosis worldwide distributed reaching hyperendemic proportions in Brazil. Many isolates from patients with sporotrichosis are preserved in culture collections by different methods around the world. The preservation methods are used to maintain the viability and the morphophysiological and genetic characteristics of isolates for long periods. In this study, we evaluated 34 isolates, previously, identified as *S. schenckii* by a classical identification method, initially preserved by periodical subcultures and then under mineral oil at culture collection of Oswaldo Cruz Institute/Fiocruz, to re-identify them by polyphasic identification. Our results showed that seven isolates remained viable for 34 to 64 years under oil, one isolate lost the ability to sporulate which was reverted by using a medium culture supplemented with rosebush branches and all of them were identified as *Sporothrix schenckii sensu stricto* by morphological, physiological, partial β-tubulin gene sequencing and phylogenetic analysis.

## Highlights

• Impact of mineral oil as a method of preservation on clinical isolates of *Sporothrix schenckii*.
• *Sporothrix* spp sporulation induction using a culture medium supplemented with rose bush branches.
• Usefulness of polyphasic methodology in the re-identification of species of *Sporothrix schenckii* complex.

## Graphical abstract

**Figure:**
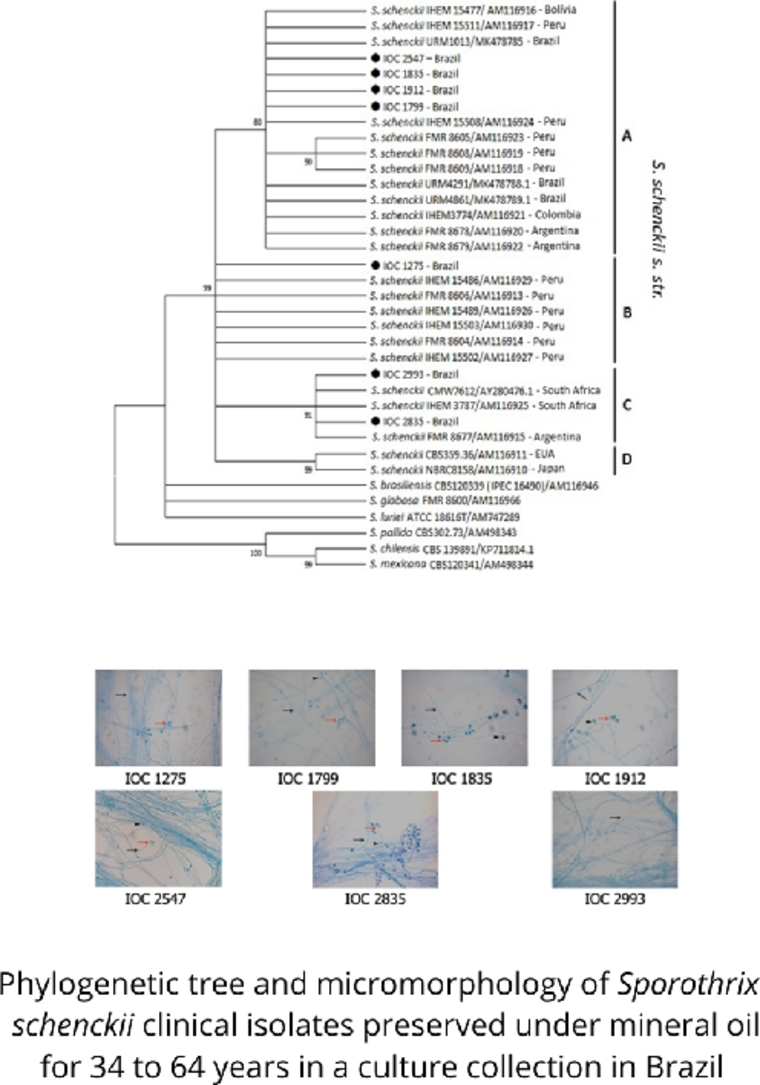

## 1 Introduction

*Sporothrix* species are thermodimorphic pathogenic fungi that cause human and animal sporotrichosis (Marimon et al., 2007; Oliveira et al., 2011; Rodrigues et al., 2016), a subcutaneous mycosis with a worldwide distribution that reaches hyperendemic proportions in some regions in Brazil (Chakrabarti et al., 2015; Gremião et al., 2021).

Since the zoonotic transmission of sporotrichosis was evidenced (Read and Sperling, 1982), this infection proved to be more relevant and consequently, its etiological agents have been more studied (Gremião et al., 2017). For this reason, the *in vitro* maintenance and preservation of these fungal isolates, ensuring the viability and morphological and physiological stability, become essential for future taxonomic, diagnostic, and treatment studies.

Fungal isolates are preserved in culture collections and the storage time varies around 10 years or less, depending on the species being preserved and the storage method (Nakasone et al., 2004). Culture collections are conservation centers for *ex situ* genetic resources whose main objectives include the preservation and supply of biological material and associated information for scientific and industrial research and development (Onions et al., 1983). The preservation methods have the purpose of maintaining the viability and the morphological, physiological and genetic characteristics of the isolates (Onions et al., 1983; Borba et al., 1992; Borba and Rodrigues, 2000; Cavalcanti et al., 2013).

Due to the great biological diversity of the fungi, several methods of preservation are necessary to ensure the viability and the phenotypic and biochemical stability of the cultures over time (Nakasone et al., 2004).

The choice of the best preservation method for each fungal species used for periodic monitoring to verify its morphology, pathogenicity and genetic stability is required to avoid or minimize the problems related to storage conditions. In addition, it is important to take into account the characteristics of the method, maintenance costs, the structure of the collection, and availability of equipment, among other factors (Ryan et al., 2000; Freitas et al., 2019).

One of the most important collections of fungal cultures in Brazil is the Culture Collection of Filamentous Fungi of Oswaldo Cruz Institute/Fiocruz (CCFF-IOC – available on website http://ccff.fiocruz.br/index?catalog), which is composed of strains of filamentous fungi from different taxonomic groups preserved by sterile mineral oil, lyophilization, and cryopreservation. Among the several preserved species, there are isolates identified as *Sporothrix schenckii* by classical identification, maintained since the 1920s, initially by periodical subcultures and later under mineral oil. At that time, the *Sporothrix schenckii* complex had not yet been described and new species have only emerged in the late twentieth century (Rodrigues et al., 2020). Therefore, it becomes important to evaluate/re-identify the *Sporothrix schenckii* preserved at CCFF-IOC over time by new methodologies of identification.

The method of fungal preservation under sterile mineral oil was first used by Sherf (1943) and its effectiveness has been demonstrated in several studies of *Sporothrix* spp. preservation (Borba et al., 1992; Lima and Borba, 2001; Lima et al., 2003) and of other fungi (Fennel, 1960; Onions, 1971; Barnes, 1984; Schonborn, 1989). This method reduces the metabolic activity of organisms and decreases the dehydration of the culture medium (Ajello et al., 1951; Fennel, 1960; Borba et al., 1992). It has low cost, may be applied in any laboratory, and does not require specialized equipment. However, extreme care must be taken because high layers of oil on the fungal colony may cause deleterious effects and irreversible changes of the fungal isolate (Lima and Borba, 2001).

The aim of this study was to evaluate the viability and *in vitro* stability of the morphological and physiological pattern of isolates, previously identified as *S. schenckii*, preserved for long periods of time under mineral oil, to re-identify them by new methodologies (polyphasic identification).

## 2 Material and methods

### 2.1 Fungal isolates and culture conditions

Thirty-four isolates, identified by classical identification as *Sporothrix schenckii* when deposited in the Culture Collection of Filamentous Fungi of Oswaldo Cruz Institute, Fiocruz (CCFF-IOC) were used in this study. These isolates were maintained, at room temperature, initially by successive subcultures and between 1948 and 1949 they were transferred to tubes containing potato dextrose agar (PDA) medium and covered with sterile mineral oil.

### 2.2 Morphological studies

The isolates were removed from the mineral oil, cultured on Difco™ potato dextrose agar - PDA (Becton, Dickinson and Company, Sparks, USA), and maintained at room temperature. After growth, they were transferred to PDA and kept at 30 °C for 21 days for the macromorphological analysis of the colonies.

Microcultures (Rivalier and Seydel, 1932) using PDA were performed to examine the morphological structures and sporulating ability of the isolates. In addition, the isolates were cultured on Difco™ corn meal agar – CMA, to determine the presence of pigmented conidia. All the experiments were conducted for 12 days at 30 °C (Marimon et al., 2007).

Isolates that showed no sporulation were transferred to Petri dishes containing Difco™ malt extract agar – MEA supplemented with sterilized plant tissues as rose bush branches (adapted from Borba and Rodrigues 2000).

### 2.3 Physiological studies

The growth rate at 30 °C and 37 °C of the isolates was determined on PDA as described by Oliveira et al. (2011). The diameter of the colonies (mm) was measured on the 21st day.

The dimorphic process (mycelial to yeast forms) was evaluated by subculturing the isolates on Difco™ brain heart infusion – BHI, added with agar at 37 °C. Subcultures were done each seven days for four times to obtain the yeast-like phase. Cells were collected and prepared in Amann lactophenol-cotton blue (TCS Biosciences Ltd, Buckingham, United Kingdom) and were monitored by microscopy (Marimon et al., 2007).

The carbohydrate assimilation test was performed on a 96-well polystyrene microtiter plate, sterile, containing Difco™ Yeast Nitrogen Base culture medium – YNB, plus carbohydrate sources such as 0.5% glucose (used as positive control), 0.5% sucrose, and 0.5% raffinose (Marimon et al., 2007). The plates were incubated at 25 °C for 10 days taking two readings, after 5 and 10 days. The criterion of a positive or negative reaction was based on a visual evaluation of the growth of the isolates. The presence of fungal growth was considered positive and the absence was negative (Marimon et al., 2007; Oliveira et al., 2011).

### 2.4 Molecular identification

Genomic DNA extraction from isolates was performed by the chloroform/isoamyl alcohol method (Oliveira et al., 2011).

For molecular identification, partial β-tubulin genes (BT2) were amplified using the following primers: BT2-F (5´GG[CT]AACCA(AG)AT(ATC)GGTGC(CT)GC(CT)3`) and BT2-R (5´ACCCTC(AG)GTGT AGTGACCCTTGGC3`), from primers Bt2a and Bt2b described by (Glass and Donaldson, 1995). The PCR conditions used for BT2 amplification were similar to those previously described by Marimon et al. (2006). For each reaction, we added 25 ng of DNA template and a 10 µM concentration of each primer in a total volume of 25 µL. The amplification program included 35 cycles and an annealing temperature of 60 °C. Following PCR, amplicons were purified with QIAquick PCR purification kit (Qiagen, Valencia, USA) and sequenced using BigDye™ Terminator v.3.1 cycle sequencing kit (Applied Biosystems by Thermo Fisher Scientific, Massachusetts, USA) and capillary electrophoresis (96 capillaries) – 3730xL – RPT01A (Applied Biosystems™ Genetic Analyzers, Thermo Fisher Scientific) in the Sequencing Platform at Oswaldo Cruz Foundation.

The sequences were edited in CodonCodeAligner (http:www.codoncode.com/aligner), then the identity of our nucleotide sequences was verified by BLASTN (Basic Local Alignment Search Tool- NIH) search (http:\\www.ncbi.nlm.nih.gov/blast). Several published BT2 sequences from *Sporothrix* species were retrieved from NCBI GenBank S. *brasiliensis* CBS120339 (formerly IPEC 16,490), *S.* *globosa* FMR 8600, *S.* *luriei* ATCC 18616T, *S.* *palida* CBS302.73, *S. chilensis* CBS 139,891, *S. mexicana* CBS 120,341, *S*. *schenckii* (FMR 8604; FMR 8605; FMR 8606; FMR 8608; FMR 8609; FMR 8677; FMR 8678; FMR 8679; IHEM3774; IHEM 3787; IHEM 15,502; IHEM 15,503; IHEM 15,508; URM4291; URM1013; URM4861; IHEM 15,511; IHEM 15,477; IHEM 15,486; IHEM 15,489; CMW7612; CBS359.36; NBRC8158) and sequences from both DNA strands were generated, edited with the Sequencher ver. 4.6 software package (Genes Codes Corporation, USA), and aligned by means of the MEGA X software (Kumar et al., 2018).

All phylogenetic analyses were performed based on a method previously described by Tamura et al. (2004), the multiple nucleotide sequence alignment was inspected, visually adjusted and subsequently used for neighbor-joining analysis (Saitou et al., 1987) performed using MEGA X software (Kumar et al., 2018) (http://www.megasoftware.net/), and the phylogenetic relationships among isolates were evaluated from tree topologies by Maximum Parsimony (MP) algorithm (Saitou et al., 1987), confidence was estimated using the Bootstrap test (Felsenstein et al., 1985) with 1000 replicates.

## 3 Results

### 3.1 Viability and physiological characterization of the isolates

Table 1 summarizes the data from the analysis of the isolates. Regarding the viability, of the 34 isolates from the CCFF-IOC seven were recovered (20.6%). The storage time in mineral oil ranged from 64 to 34 years, according to the information in the CCFF-IOC catalog sheets (Table 1). The viable isolates were originally isolated in Rio de Janeiro/Brazil, prior to the zoonotic epidemic in this city, from lesions of patients with sporotrichosis, except for IOC 1799 clinical isolate, which was a sample of Japanese origin.

**Table 1:** Summary of the characteristics (morphological and physiological) of *Sporothrix* spp. recovered from the Culture Collection of Filamentous Fungi of Oswaldo Cruz Institute, Fiocruz.

No. Isolates	Year of entry into CCFF-IOC	Period under mineral oil (years)	Presence of pigmented conidia	Colony diameter in BDA at 21 days (mm)		Thermo-conversion in BHI medium at 37 °C	Carbohydrateassimilation	
				30 °C	37 °C		Sucrose	Rafinose
IOC 1275	1929	35	No	29	6	Yes	+	+
IOC 1799	1935	59	No	27,5	6,5	Yes	+	+
IOC 1835	1936	34	No	31,5	8,5	Yes	+	+
IOC 1912	1945	56	Yes	34	4,5	Yes	+	+
IOC 2547	1948	64	No	35	9	Yes	+	+
IOC 2835	1950	34	No	33	10,5	Yes	+	+
IOC 2993	1951	37	No	24,5	8,5	Yes	+	+

+ (presence), - (absence).

All the studied isolates grew better at 30°, produced non-pigmented conidia (except for IOC 1912 isolate), were able to convert from mycelial to yeast forms, and assimilated all the sources of carbohydrates. Only IOC 2993 isolate showed slower conversion when compared to the others and produced yeast-like cells after 28 days of incubation.

### 3.2 Morphological characterization

**Fig. 1:**
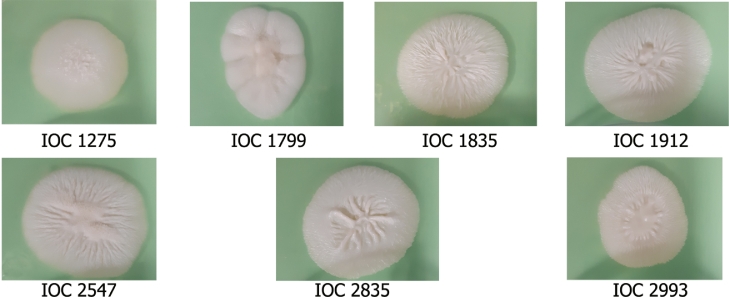
Macromorphological analysis of *Sporothrix* spp. after 21 days of incubation on PDA at 30 °C. **A** – IOC 1275; **B** – IOC 2993; **C** – IOC 1799; **D** – IOC 1835; **E** – IOC 1912; **F** – IOC 2547; **G** – IOC 2835.

The filamentous form of all recovered isolates showed white colonies with a rough surface (Fig. 1).

**Fig. 2:**
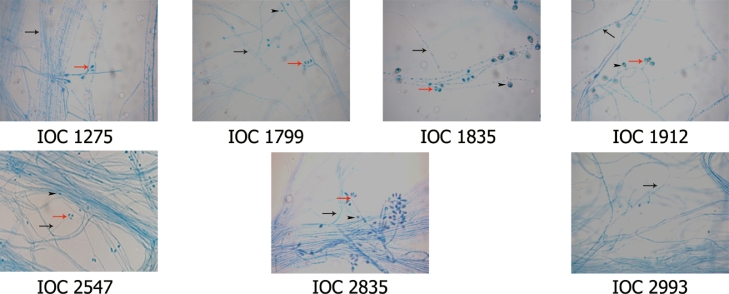
Micromorphological analysis of *Sporothrix* spp. after microculture for 12 days on PDA at 30 °C. The isolates IOC 1275, IOC 1799, IOC 1835, IOC 1912 and IOC 2835 showed sessile conidia (arrowhead) and septate hyaline hyphae (black arrow) containing conidiophores with conidia arranged in the shape of a daisy (red arrow). Isolate IOC 2993 presented only septate hyaline hyphae (black arrows). (1000X magnification).

**Fig. 3:**
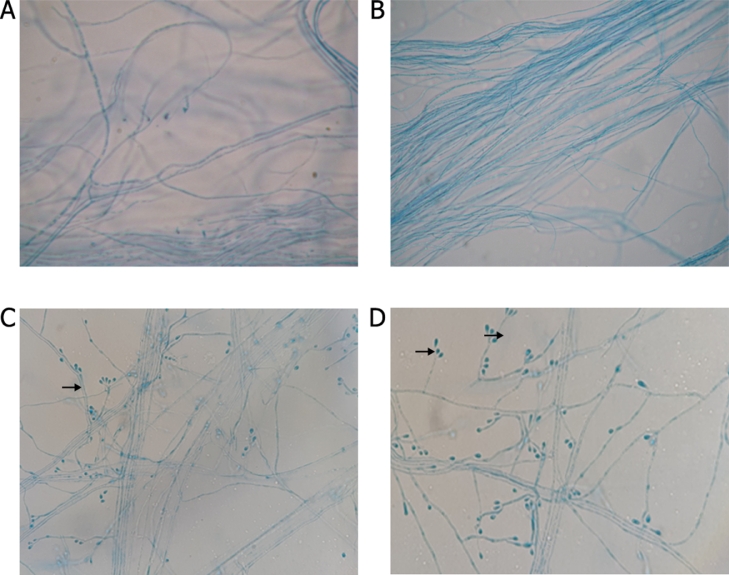
Microculture of *Sporothrix* spp. IOC 2993 for 12 days at 30 °C. **A** and **B** show the isolate cultured on PDA producing only thin, hyaline hypha. **C** and **D** show the isolates cultured on MEA with rose bush branches presenting sessile conidia and thin, septate hyaline hypha containing conidia arranged in the shape of a daisy at the end of conidiophores (Arrows) (1000X magnification).

Microcultures revealed in all isolates thin, hyaline, septate, and branched hyphae. All of them sporulated, presented oval or pyriform conidia, grouped in the shape of a daisy at the end of the conidiophores, characteristic of the *Sporothrix schenckii* complex, with the exception of isolate IOC 2993 (Fig. 2). However, after transferring it to MEA with rosebush branches, this isolate was able to produce conidia (Fig. 3).

### 3.3 Molecular identification

The amplified BT2 genes yielded DNA fragments of approximatively 410 bp in size. GenBank search revealed that these isolates showed a similarity of 98 - 100% to the *β-tubulin* sequences of *S. schenckii sensu stricto*. For that, a BLAST program was used by submitting the nucleotide sequences of our seven isolates and comparing them with sequences from *Sporothrix* species deposited in the NCBI GenBank.

All the sequences from the isolates studied were deposited in the GenBank database under accession numbers OK318447 to OK318453.

**Fig. 4:**
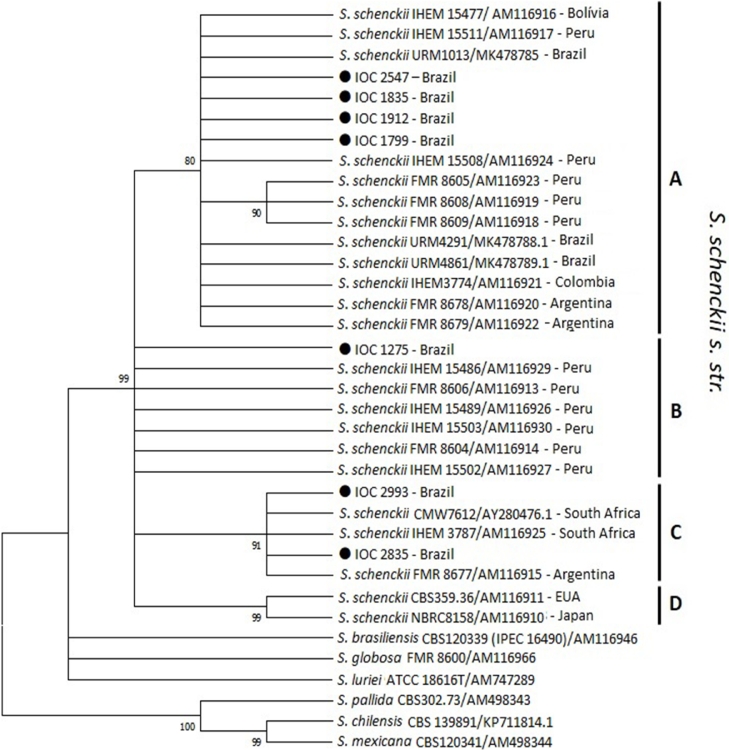
Phylogenetic relationships between the isolates IOC 2547, IOC 1835, IOC 1912, IOC 1799 IOC 1275, IOC 2993 and IOC 2835 with reference strains of the *Sporothrix schenckii* complex inferred from β-tubulin sequences by Neighbor-Joining method [27]. The optimal tree is shown. The percentages of replicate trees in which the associated taxa clustered together in the bootstrap test (1000 replicates) are shown next to the branches [28]. The evolutionary distances were computed using the Maximum Composite Likelihood method [26] and are in the units of the number of base substitutions per site. This analysis involved 31 nucleotide sequences. There were a total of 428 positions in the final dataset. Evolutionary analyses were conducted in MEGA X [25]. Bootstrap support values above 80% are indicated at the nodes.

In order to ensure the re-identification and know the phylogenetic relationship of the re-identified CCFF-IOC isolates with *Sporothrix schenckii* complex isolates described in the methodology, a phylogenetic tree was inferred. The phylogenetic tree grouped the IOC isolates into three (A – IOC 2547, IOC 1835, IOC 1912, IOC 1799; B – IOC 1275; C – IOC 2993, IOC 2835) of four clades formed (Fig. 4).

## 4 Discussion

Of the thirty-four isolates from the Culture Collection Filamentous Fungi of Oswaldo Cruz Institute, Fiocruz, seven were viable after preservation for a long time under mineral oil. It is reasonable to assume that 27 isolates did not resist the conditions of microaerobiosis, lack of nutrients, and accumulation of toxic metabolites produced during the preservation processes (Fennel, 1960). Borba et al. (1992) studying *S. schenckii* isolates from the same culture collection 29 years ago obtained a recovery rate of 85% with macro and microscopic characteristics unchanged. Subsequently, Lima and Borba (2001), 9 years later, confirmed these data, but some isolates from the study had their morphology, ability to sporulate and perform the dimorphic process compromised. In this work, the same isolates studied by the aforementioned authors were evaluated from the original tubes demonstrating that the additional time to which the isolates were submitted greatly compromised their viability.

Although this method of preservation has the advantages of being simple and requiring few resources, there are disadvantages, such as the need for frequent monitoring of fungal colonies and the relative and laborious frequency of subculturing. Furthermore, there are reports of changes in the biochemical activity, morphological stability, and virulence profile of *S. schenckii* after storage (Borba et al., 1992; Lima and Borba 2001; Lima et al., 2003, 2004). Moreover, Mendes da Silva et al. (1994) observed that this method of preservation is not as efficient for other dimorphic fungi, such as *Paracoccidioides brasiliensis*.

The morphological and physiological characteristics of the isolates studied here are in agreement with those reported by Marimon et al. (2007). The diameter of all colonies of the isolates did not exceed 50 mm at 30 °C, compatible with *S. schenckii* complex (Marimon et al., 2007)*.* However, this is just one of the tools used to identify species in the *S. schenckii* complex. Another parameter to be considered in the characterization of this complex is the morphology and color of conidia (Marimon et al., 2007). Only IOC 2993 isolate did not produce the typical reproductive structure that identifies it. But, the use of a natural supplement to the culture medium, as described by Borba and Rodrigues (2000) for the recovery of Coelomycetes, was able to stimulate the production of conidia in typical structures of the species, facilitating the morphological authentication of this isolate.

All isolates produced unpigmented conidia, with the exception of IOC 1912. Although this isolate is one of those that remained for the longest time under mineral oil (56 years) it still produced pigmented conidia. It is known that the long period of preservation under mineral oil may cause changes, some of them irreversible, such as the pigmentation ability of the conidia and also the performance of dimorphism (Lima and Borba 2001; Lima et al., 2003).

In relation to thermotolerance, all isolates in this study produced cigar-shaped budding yeasts when cultured at 37°C. IOC 2993 isolate performed a slower conversion from mycelial to yeast form, probably due to the prolonged stress of the preservation process as described in the literature (Borba et al., 1992; Lima and Borba 2001; Lima et al., 2004)_,_ but it was able to turn into yeast-like cells. Lima et al. (2004) showed irreversible alterations in dimorphic fungi such as *Blastomyces dermatitidis* and *Paracoccidioides brasiliensis* preserved in mineral oil even after *in vivo* passage, in contrast to S. *schenckii* isolates that demonstrated greater resistance. These authors affirm that the mechanisms of resistance that permit fungal cells to survive and to re-establish activity after long periods of preservation are not well understood and require more investigation. Some years ago, Borba et al. (2005) performing biochemical, morphological, and molecular approaches for comparing typical and atypical strains of *P. brasiliensis* after preservation under mineral oil, concluded that, undoubtedly, the maintenance under mineral oil for long periods of time altered the dimorphic process of some strains.

All isolates assimilated sucrose and raphinose carbohydrates as described by Marimon et al. (2007), who reported members of the *S. schenckii* complex. The results obtained here (morphology and physiology) were not conclusive for the identification of the isolates showing the need to use polyphasic identification to elucidate the species. Then, the molecular analysis using partial β-tubulin gene sequencing identified all IOC isolates as *S. schenckii sensu stricto*.

The phylogenetic relationship of CCFF-IOC isolates studied here with other *S. schenckii* isolates from several geographic regions inferred by tree-based on BT2 sequences demonstrated intraspecific diversity between them and grouped the isolates inside clusters described by Marimon et al. (2006). Four isolates (group A) joined the isolates belonging to cluster IIa and three (group B and C) to cluster IIb described by Marimon et al. (2006). It is interesting to remember that other studies using different methodologies have already demonstrated intraspecific diversity in this species (Marimon et al., 2007; Zhou et al., 2013; Rodrigues et al., 2014; Sasaki et al., 2014; Zhang et al., 2015).

Few studies have been carried out in order to analyze *Sporothrix* species preserved in collections, mainly those isolated and identified as *S. schenckii* before the sporotrichosis epidemic in Rio de Janeiro, Brazil. Therefore, our objective joins the study done by Rodrigues et al. (2013) that re-examined, by molecular methods, the identification of members of a large research collection of isolates from sporotrichosis patients in Brazil classified as *S. schenckii sensu lato*, showing its valuable contribution to the improvement of collections.

## 5 Conclusion

Based on the results described here and those obtained by other authors (Borba et al., 1992; Lima and Borba 2001; Lima et al., 2003, 2004) over the years, although *Sporothrix* species are considered more resistant to the mineral oil method, we do not recommend it because of the increased risk of alterations and also because it requires laborious surveillance to guarantee that fungal isolates are well preserved. As seen in this study we used media supplemented to induct the sporulation suggesting the use of rose bush branches as a fungal recuperation method in culture collections. Finally, the BT2 region has been successfully used by many authors (Marimon et al., 2006; Zhou et al., 2013; Freitas et al., 2015; Rodrigues et al., 2016; Florez-Munoz et al., 2019) in the identification of species of the *Sporothrix schenckii* complex and in this study, we could show its convenience in the identification of CCFF-IOC isolates proving to be a powerful tool to use in culture collections. Recently, Valeriano et al. (2020) used this same region to perform a taxonomic review of *Sporothrix* species stored at Micoteca URM, Recife, Brazil, previously identified as *S. schenckii* by classical methods of taxonomy and re-identified four isolates as *S. chilensis*. We believe that studies with *Sporothrix* species from culture collections, as well as other microorganisms, are of invaluable importance due to the emergence of new species in this complex as well as the increase of new cases of sporotrichosis.

## CRediT authorship contribution statement

**Thais Barreira:** Methodology, Investigation, Formal analysis, Writing – original draft. **Danielly Corrêa-Moreira:** Supervision, Visualization, Writing – review & editing. **Cintia Borba:** Conceptualization, Writing – review & editing. **Aurea Moraes:** Visualization, Writing – review & editing. **Manoel Oliveira:** Conceptualization, Resources, Supervision, Project administration, Funding acquisition, Writing – review & editing.

## Declaration of Competing Interest

The authors declare that they have no known competing financial interests or personal relationships that could have appeared to influence the work reported in this paper.
